# Case report: Acute liver failure in children and the human herpes virus 6-? A factor in the recent epidemic

**DOI:** 10.3389/fped.2023.1143051

**Published:** 2023-04-26

**Authors:** Suz Warner, Rachel M. Brown, Gary M. Reynolds, Zania Stamataki, Deirdre A. Kelly

**Affiliations:** ^1^ Centre for Liver and Gastrointestinal Research at the Institute of Immunology and Immunotherapy, University of Birmingham, Birmingham, United Kingdom; ^2^ The Liver Unit, Birmingham Children’s Hospital, Birmingham, United Kingdom; ^3^ Cellular Pathology, Queen Elizabeth Hospital, Birmingham, United Kingdom; ^4^ School of Immunity and Infection, College of Medical and Dental Sciences, University of Birmingham, Birmingham, United Kingdom

**Keywords:** acute liver failure, pediatric, hepatitis, human herpes virus 6 (HHV-6), and liver transplant

## Abstract

The 2022 worldwide epidemic of acute hepatitis and liver failure in young children has led to a focus on unusual causes for childhood acute hepatitis. In the UK epidemic, human herpes virus subtype 6B (HHV-6B) was detected along with adenovirus subtype-41F in severely affected children, especially in those requiring liver transplantation (LT). The lifting of COVID lock-down measures has coincided with the rise in these common childhood infections with a higher than expected rate of systemic complications. The sudden exposure of young children to common childhood infections from which they were protected during the pandemic may have induced an abnormal immune mediated response potentiated by multiple pathogen exposure. Primary HHV-6 infection is one such common childhood infection. Classically known as Roseola infantum due to the appearance of a widespread erythematous rash on fever subsidence (exanthema subitem), it has a peak incidence of 6–12 months of age and almost all children will have been infected by age 2. It is the virus most frequently associated with febrile convulsions but the more serious complications of hepatitis and liver failure are rare. We report on the historic cases of three female infants who had suspected primary HHV-6B infection, acute hepatitis and rapid progression to acute liver failure (ALF) requiring LT. Appearances of their native liver were identical to those described in children in the recent hepatitis epidemic. Deteriorating clinical trajectories of recurrent graft hepatitis and rejection-like episodes followed and all three succumbed to graft failure with HHV-6B detected posthumously in their liver allografts. Our case series and the serious complications observed with the recent rise in common childhood infections is a reminder that these routinely encountered pathogens can be deadly especially in the young immunologically untrained. We advocate for HHV-6 to be screened for routinely in children with acute hepatitis and the use of effective HHV-6 anti-viral prophylaxis to prevent recurrence post-transplant.

## Introduction

The dramatic rise of acute hepatitis and liver failure (ALF) in young children in 2022 has led to an unprecedented epidemic, with over 1,000 cases reported worldwide (1). In the latest UKHSA technical briefing, 260 UK children (median age 3 years) had acute hepatitis of unknown aetiology since January 2022 (2). Although no deaths were reported and most recovered with conservative management, 12 required liver transplantation (LT). The majority of patients had Adenovirus subtype-41F identified but no adenovirus inclusions were observed in the native liver, leading to the speculation that a secondary virus may be involved in those with severe disease or required a LT (3).

Secondary viruses, other than adenovirus, has been detected on screening (2).

In 13 UK cases of the recent hepatitis epidemic, blood and/or liver samples sent for metagenomic analysis all tested positive for human herpes virus 6 (HHV-6) (2).

Human herpes viruses 6 (HHV-6) is a common childhood infection and almost all children will have been infected by age 2 years (4, 5). Systemic involvement is rare but consists of acute hepatitis, liver failure, pneumonitis, encephalitis and bone marrow suppression (4, 5). HHV-6B is the more prevalent subtype and is linked not only to acute hepatitis and liver failure but also to rejection and graft failure in children (4–6). The hepatitis epidemic is hypothesized to be related to the lifting of COVID lock-down measures, resulting in the exposure of young children to common childhood infections such as adenovirus and HHV-6 from which they were protected during the pandemic.

We report on the clinical course of three female infants with acute hepatitis and ALF who progressed to LT, in whom primary HHV-6 infection was suspected. All followed a deteriorating clinical trajectory of recurrent graft hepatitis and rejection-like episodes. All succumbed to graft failure with HHV-6B detected in their liver allografts.

Our case series and the serious complications observed with the recent rise in common childhood infections is a reminder that these routinely encountered pathogens can result in significant morbidity and mortality, especially in the young immunologically untrained.

Co-infection with HHV-6 and adenovirus may pertain to a more aggressive phenotype in susceptible children and lead to the development of ALF as observed in the hepatitis epidemic.

## Case series

Three female infants (aged 6–11 months) presented with acute hepatitis of unknown cause which rapidly deteriorated within days to acute liver failure (ALF) (Table 1). All were previously well children who had a short prodromal coryzal illness. Two had a transient maculopapular erythematous rash. Screening to investigate for underlying liver disease excluded inborn errors of metabolism, autoimmunity and drug induced liver injury.

**Table 1:** Patient characteristics and clinical progression.

Patient demographics and clinical characteristics	Findings n=
Age at ALF presentation	6 to 11months
Gender	All female
Prodromal illness with URTI	3
Widespread erythematous blanching rash	2
HHV-6 viraemia pre-transplantation*	2
Progression to liver transplantation	3
Aciclovir prophylaxis	3
Immunosuppression:	
Basiliximab induction, maintenance Tacrolimus	2
Dacluliximab induction, maintenance Tacrolimus & Prednisolone	1
Graft dysfunction at 6-8 weeks post-LT	3
ACR diagnosed in liver allograft biopsy**	3
Immunosuppression used to treat ACR:	
Methylprednisolone	3
Basiliximab	3
Tacrolimus & Prednisolone	3
Mycophenolate mofetil (MMF)	2
Persistance of HHV-6 viraemia	3
Progression to chronic rejection, graft failure and re-transplantation	3
HHV-6 detected in allografts	3

* Patient 1 had blood sent for HHV-6 screening but this was not processed; her allograft biopsy at 6weeks post-LT had HHV-6 detected. All subsequent blood tests were positive for HHV-6.

** ACR for all 3 patients were diagnosed in their liver allograft biopsy at 6-8 weeks post-LT and subsequent allografts, coinciding with episodes of HHV-6 viraemia and graft dysfunction. ALF, acute liver failure; URTI, upper respiratory tract infection; HHV-6, human herpes virus 6; LT, liver transplantation; ACR, acute cellular rejection.

The only positive virology detected pre-transplant was HHV-6 PCR on whole blood from two patients; quantification was not performed at the time. Blood was sent for HHV-6 screening in the third patient but this was not processed. She was HHV-6 PCR positive on subsequent blood tests. All three underwent super urgent listing and progression to liver transplantation (LT). Histology of their native livers showed acute hepatitis with submassive hepatic necrosis with no specific diagnostic features identified. There were no viral inclusions. There was no evidence of fibrosis. Appearances in their native livers were however, identical to those described in the recent outbreak termed “acute hepatitis of unknown aetiology in children”.

Their induction and maintenance immunosuppression is shown in Table 1. They all received protocol prophylactic aciclovir for a 3 month duration.

**Figure 1:**
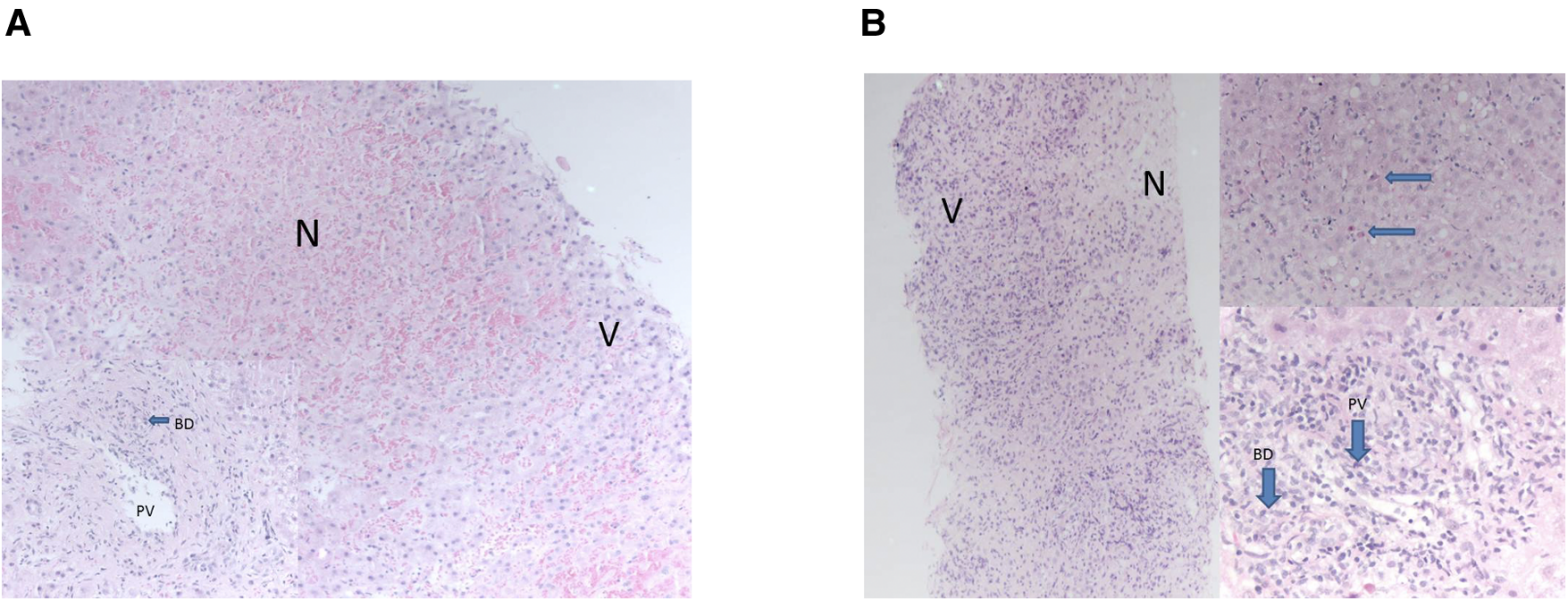
(**A**) areas of necrosis were seen “N” contrasting with nucleated viable hepatocytes “V”. Portal tracts demonstrated bile duct damage “BD” and endothelitis of portal vein branches “PV”—features of acute rejection. The necrosis was only seen in this one biopsy. (**B**) Necrosis “N” was seen at one end of the biopsy, “V” marks viable cells with nuclei. Viable parenchyma showed features of acute hepatitis with acidophil bodies, top right, arrows. Portal tracts showed severe endothelitis in portal veins “PV” and severe inflammatory bile duct damage “BD”—features of acute rejection. These changes, of necrosis and acute hepatitis, were only seen in this one biopsy of many taken from all allografts.

All three developed graft hepatitis at 6 weeks post-LT along with HHV-6 viraemia; histology of their allograft biopsies appeared consistent with acute cellular rejection (ACR) which required pulse methylprednisolone treatment along with an increase of their maintenance IS (Table 1). The liver biopsies taken on each occasion of graft hepatitis were suggestive of acute cellular rejection and patients were treated accordingly with escalation of immunosuppression. Histological features outwith the spectrum recognised in rejection were noted in the allografts (Figures 1A,B); patient 2 had acute haemorrhagic necrosis in her first allograft and patient 3 had an area of superimposed acute hepatitis of possible viral origin in her second allograft. Repeat viral hepatitis screen performed at the time confirmed persistent HHV-6 viraemia in both. The same trajectory of recurrent ACR, chronic rejection and graft failure followed despite immunosuppression escalation.

Patient 1 had a recurrence of graft hepatitis at 16 months post-LT. Repeat viral screening confirmed HHV-6 viraemia. Very high viral titres were detected (maximum 7 × 10^6^ copies/ml) which led to the suspicion of inherited viral chromosomal integration (ciHHV-6), a unique feature of HHV-6. She was placed on a month's course of oral Valganciclovir based on local virology expert advice which saw a decline but not resolution of the HHV-6 viraemia; ciHHV-6 was ruled out on PCR testing of the allograft. Despite treatment for the ACR and antivirals, chronic rejection and decompensated graft failure ensued. She was listed for a second-LT but unfortunately did not survive the second transplant due to overwhelming sepsis.

Patient 2 followed the same trajectory of recurrent ACR, chronic rejection and graft failure. She progressed to a second-LT but her post-transplant recovery was complicated with CMV hepatitis and HHV-6 coinfection. Her blood CMV levels became undetectable with systemic Ganciclovir treatment but the HHV-6 viraemia persisted. She again had recurrent ACR, chronic rejection and unfortunately acute graft decompensation supervened and the patient died 2 years on from her initial ALF presentation. HHV-6 viraemia was persistent throughout.

Patient 3 had a more complicated post-LT course. She had moderate EBV viraemia which decreased with IS reduction. ACR was diagnosed at 6 weeks post LT. She had a pancytopenia around this time but her bone marrow aspirate showed reactive marrow changes only. Marrow and graft function improved but cholestasis remained and imaging revealed a biliary anastomotic stricture; this resolved with surgical revision. An allograft biopsy taken at the time of surgery showed extensive ductopenia consistent with chronic rejection. Graft function deteriorated in the coming months and she progressed to a second- LT.

Her EBV levels were negligible (<500 copies/ml) but post-transplant lymphoproliferative disease (PTLD) was found in her first allograft explant. She responded well to treatment with Rituximab and immunoglobulin support and had no residual PTLD demonstrated.

Graft hepatitis with ACR biopsy changes occurred at 8 weeks post second-LT and despite IS escalation, graft failure developed in a matter of months. She went on to have a third LT. There was no evidence of PTLD in the second allograft. A repeat viral hepatitis screen showed a recurrence of HHV-6 viraemia. Gancyclovir was commenced followed by the addition of Foscarnet but the patient deteriorated rapidly with sepsis and multi-organ failure and died 14 months on from the onset of the ALF.

**Figure 2:**
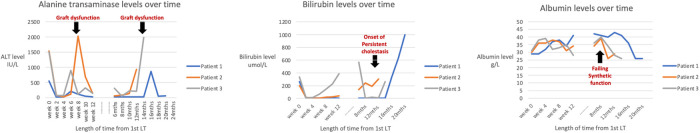
Line graphs of the alanine transferase (ALT), bilirubin and albumin levels at time points of “graft dysfunction”, onset of persistent cholestasis and falling albumin levels for each patient.

Table 2 shows the HHV-6 DNA quantification results of the liver allografts and Figure 2 shows timepoints of graft dysfunction, onset of persistent cholestasis and falling albumin levels for our patients in their post-transplant course. From a viral screen perspective, Patient 2 and 3 had persistent blood HHV-6 PCR positivity but unfortunately quantification was not performed and no blood samples were available for further analysis. Patient 2 had CMV hepatitis post 2^nd^ LT and Patient 3 had moderate EBV viraemia post 1^ST^ LT as described above. No other concomitant viruses were detected aside from HHV-6 throughout their pre and post-transplant courses. An underlying primary immunodeficiency may be masked by iatrogenic immunosuppression and therefore T cell subsets, immunoglobulin profiles and vaccine responses were carried out for Patient 1. Results of these were all within the normal range. Patients 2 and 3 were historical cases in which no blood samples were available for analysis. Baseline immunoglobulin levels for all 3 patients were within normal limits.

### HHV-6 detection in liver

HHV-6 immunohistochemistry staining on the native livers were inconclusive; non-specific macrophage and hepatocyte anti-HHV-6 staining was detected but not to a significant level. These findings were consistent to that described in the current epidemic liver immunostaining.

In view of the clinical similarities and positive virology for HHV-6 in blood, archived frozen samples of allograft liver tissue from each patient were tested for HHV-6 by qPCR. The results confirmed HHV-6 subtype B in the allografts of all three patients, suggesting a recurrent HHV-6B graft infection following primary HHV-6 infection and ALF (Table 2).

**Table 2:** Post-humous testing of liver explants and allograft biopsies for HHV-6 DNA.

Patient No.	Graft dysfunction	Timeout from LT	Histology report	HHV-6 DNA detected in liver allograft biopsy	Liver allograft HHV-6 quantification (copies/ml)	Liver tissue section type
1	Feb 2015	6 weeks	ACR	Yes	2,92,270	1st Allograft Biopsy
	Feb 2016	12 months	ACR	Yes	95,790	1st Allograft biopsy
	July 2016	16 months	Chronic rejection	Yes	64,670	1st Allograft biopsy
2	Jan 2014	6 weeks	ACR	No	Negative	1st Allograft biopsy
	May 2014	6 months	ACR	Yes	2440	1st Allograft biopsy
	Nov 2014	6 weeks	ACR	Yes	1480	2nd Allograft biopsy
3	Oct 2006	6 weeks	ACR	Yes	3090	2nd Allograft biopsy
	Nov 2006	15 weeks	Chronic rejection	Yes	4640	Explant of 2nd allograft
	Feb 2007	12 weeks	ACR	No	Negative	3rd Allograft biopsy
	April 2007	5 months	ACR	Yes	90,310	3rd Allograft biopsy

LT, liver transplant; HHV6, human herpes virus 6; ACR, acute cellular rejection.

Their native liver tissue were formalin-fixed and paraffin embedded and thus not suitable for qPCR testing.

## Discussion

Human herpes virus 6 (HHV-6) is increasingly recognized as a cause of acute liver failure (ALF) in children (6, 7). The young age and phenotype described with HHV-6 infection matches our case series. Primary infection, lack of pre-existing HHV-6 antibodies and an immature immune system could account for the higher rates of systemic complications (3, 6).

Our case series and the recent hepatitis/ALF epidemic, in which adenovirus and HHV-6 have both been isolated, is a reminder that these common childhood infections can lead to significant morbidity and mortality, especially in previously unexposed young children.

The lifting of COVID lock-down measures has coincided with the rise in common childhood infections with a higher rate of systemic complications. The sudden exposure of young children to common childhood infections from which they were protected during the pandemic may have induced an abnormal immune mediated response potentiated by multiple pathogen exposure.

HHV-6 is one such common pathogen in which hepatitis and ALF, although rare, is speculated to be a factor in the 2022 acute hepatitis epidemic (2, 3). Blood and/or liver samples from thirteen children with acute hepatitis of unknown aetiology in the UK epidemic all tested positive for HHV-6 (2). Moreover, several recent reports have highlighted the association between primary HHV-6 infection and higher rates of graft hepatitis, rejection, and failure. Pischke et al. reported high intrahepatic HHV-6 viral load as an independent risk factor for decreased graft survival in young paediatric LT recipients (6, 8). This contrasts with the adult post-LT population in which HHV-6 graft failure is unusual as latent reactivation as opposed to primary infection accounts for the majority of HHV-6 graft infection in this cohort (6–8). With this in mind, co-infection with HHV-6 may have played a part in the recent hepatitis epidemic and paediatricians need to be aware of the complexities in successfully treating such patients post-transplant.

Viral replication and latent reactivation occur during the intense immunosuppression phase post-LT as observed with other opportunistic infections like CMV and EBV (5, 8). This phase coincided with the timing of ACR in our patients at 6–8 weeks. Pappo-Toledano et al. identified 4 children with primary HHV-6 infection out of 26 paediatric liver transplants; all were under 1 year, presented with fever and seronegative hepatitis, all progressed to liver failure needing transplantation and three patients had changes suggested of rejection within similar timeframes (9). Taking into account the varying complexities of our three patients' post-transplant course, the commonalities between all three were persistent HHV-6B viraemia, recurrent graft hepatitis and rejection, and importantly, HHV-6B was detected in their initial and subsequent liver allografts.

Our findings support the diagnosis of recurrent HHV-6B graft infection, possibly from circulating HHV-6B. Of the two HHV-6 subtypes (A and B), HHV-6B is the prevalent one associated with ALF and is linked to higher rates of graft dysfunction, rejection, failure and mortality in paediatric patients (5, 6, 8).

Serology screening for HHV-6 as a cause for ALF is not routinely performed as >90% of the population is infected by 2 years of age (10, 11). HHV-6 blood/plasma qPCR analysis is not routinely included as part of the viral hepatitis screen for acute liver failure in many centres. Nor is it performed in the pre-transplant virology assessment. Most histology departments will not routinely test for HHV-6 in the liver (5, 6). On review of our case series and the literature, there is strong evidence to support a change in patient management to include routine HHV-6 screening by qPCR in young children with acute hepatitis and/or ALF. Furthermore, metagenomic studies in the hepatitis epidemic has shown a strong association with Adeno-associated virus 2 (AAV2) in addition to HHV-6 and adenovirus (2). Both viruses enhance the lytic replication of AAV2 and it would therefore be judicious to test for the presence of AAV2.

Cholestasis, lymphocytic parenchymal infiltration and hepatocellular damage has been described in HHV-6 induced acute liver failure in children (6). Portal lymphocyte infiltration is a prominent histological feature as is confluent necrosis both in native liver and allograft HHV-6 infections (6, 12). Confluent necrosis in particular is associated with high HHV-6 tissue load. A recent study found HHV-6 DNA in 10/26 (38.5%) of liver biopsies taken for acute hepatitis of unknown aetiology in children, 4/10 had confluent necrosis identified in the liver of patients with high HHV-6 DNA (5). Two interesting aspects of the histology were identified in the allografts of our patients which were out-with the spectrum of acute rejection; an area of confluent haemorrhagic necrosis in one and superimposed acute hepatitis of possible viral origin in the other. HHV-6 graft infection may have contributed to these histological anomalies and potentiate or even mimic acute cellular rejection.

The histological features of HHV-6 graft hepatitis and rejection are not dissimilar (5, 6). One study reported 5/8 patients with HHV-6 induced graft hepatitis had acute rejection and three had lymphocytic infiltration (5). The allograft biopsies of our three patients all had features of acute cellular rejection with portal lymphocyte infiltration.

Lastly, there is also no consensus as regards to the treatment or prophylaxis of HHV-6. Both HHV-6A and HHV-6B are resistant to Aciclovir (5), the most common prophylactic antiviral used post-LT (5, 6). Ganciclovir, Cidofovir and Foscarnet are effective against HHV-6 in in-vitro studies (5, 7). Cidofovir is the antiviral used for treatment and prophylaxis in the current epidemic in view of the high rates of adenovirus detection in blood. We propose a change in antiviral prophylaxis and treatment in young children with acute hepatitis and/or ALF to one that is effective against HHV-6, such as Ganciclovir or Cidofovir as early treatment may prevent the development of ALF or graft re-infection.

## Conclusion

HHV-6, although a common childhood infection, may render young children more susceptible to an aggressive clinical course and lead to ALF. HHV-6 co-infection with Adenovirus has been identified as a factor in the severity of liver failure and requirement for LT in the 2022 hepatitis epidemic. HHV-6 should be screened for routinely in children with ALF and clinicians should be aware that poor systemic clearance and latent reactivation predisposes to allograft infection, rejection, and without adequate prophylaxis, an adverse impact on graft and patient survival.

## Data Availability

The original contributions presented in the study are included in the article, further inquiries can be directed to the corresponding author.
